# Case-control studies with affected sibships

**DOI:** 10.1186/1753-6561-1-s1-s29

**Published:** 2007-12-18

**Authors:** Karola Köhler, Melanie Sohns, Heike Bickeböller

**Affiliations:** 1 Georg-August-University Goettingen, Medical School, Department of Genetic Epidemiology, Humboldtallee 32, D-37073 Goettingen, Germany

## Abstract

Related cases may be included in case-control association studies if correlations between related individuals due to identity-by-descent (IBD) sharing are taken into account. We derived a framework to test for association in a case-control design including affected sibships and unrelated controls. First, a corrected variance for the allele frequency difference between cases and controls was directly calculated or estimated in two ways on the basis of the fixation index *F*_*ST *_and the inbreeding coefficient. Then the correlation-corrected association test including controls and affected sibs was carried out. We applied the three strategies to 20 candidate genes on the Genetic Analysis Workshop 15 rheumatoid arthritis data and to 9187 single-nucleotide polymorphisms of replicate one of the Genetic Analysis Workshop 15 simulated data with knowledge of the "answers". The three strategies used to correct for correlation give only minor differences in the variance estimates and yield an almost correct type I error rate for the association tests. Thus, all strategies considered to correct the variance performed quite well.

## Background

It is desirable to include related cases in case-control studies because pedigrees of multiple affected individuals have a higher expected frequency of susceptibility allele(s), leading to increased power [1]. Several methods have been proposed to test for association in case-control designs that take correlations due to IBD sharing into account [1-4]. Most of these determine correlations of related individuals based on prior kinship coefficients assuming no linkage under the hypothesis of no association. Only Slager and Schaid [4] incorporate individual identity-by-descent (IBD) estimates from previous linkage analyses. A comparison of the two strategies with respect to their power has been presented by Bourgain [5]. To integrate both strategies in one model we derive a unified framework to test for association including affected sibships and unrelated controls and apply the introduced test statistics to the candidate gene data set of Plenge et al. [6] as well as a replicate of the simulated single-nucleotide polymorphism (SNP) genome data.

## Methods

### Notation and assumptions

The study sample contains *n*_1 _cases and *n*_0 _controls (*n*_1 _+ *n*_0 _= *n*) with corresponding allele frequencies *p*_1 _and *p*_0 _and common frequency *p *under the null hypothesis of no association. There are *m *cases from sibships with at least two sibs and *n*_1 _- *m *independent cases. At the candidate locus, each individual has two alleles, *X*_*i*1 _and *X*_*i*2 _(*i *= 1,..,*n*) coded as 0/1. Usually only the genotype *X*_*i*. _= *X*_*i*1 _+ *X*_*i*2 _is known. For all individuals the affection status *y*_*i *_= 0/1 is given. The cases from families comprise *k *= 1,...,*K *sibships of size *m*_*k*_, and *z*_*i *_denotes the sibship of individual *i*. For the cases, the *X*_*ij *_values have a Bernoulli(*p*_1_) distribution. Cases from different sibships are assumed to be independent, cases from the same sibship are not independent. To describe the correlation structure between sibs we use a model from population genetics that considers a population consisting of different subpopulations based on the coefficient *F*_*ST *_and the inbreeding coefficient *F*_*IT*_. Sibships are regarded as small subpopulations and *F*_*ST *_denotes the correlation between two randomly chosen alleles of two individuals from the same sibship. Under the assumption of no population structure, correlations within sibships only arise from IBD sharing between sibs and *F*_*ST *_equals the expected kinship coefficient between two siblings. *F*_*IT *_measures the correlation of the two alleles within an individual and equals 0 under assumption of random mating and no further population structure.

### The test statistic

Based on the correlations *F*_*ST *_and *F*_*IT*_, the true variance of the numerator of the allelic *χ*^2^-test statistic can be calculated. One component is the sum of all alleles from cases of sibships $S=\sum {\;}_{i:y_{i}=1,z_{i}\in 1,...,K}X_{i.}$. Its true variance can be calculated as

$$Var(S)=p_{1}(1-p_{1})2m[1+F_{IT}+2F_{ST}((\sum {\;}_{k}m_{k}^{2}/m)-1)],$$

where the term in square brackets, in the following denoted by *γ*, is the variance inflation in comparison to the variance of the sum of alleles from independent cases. If the data set only consists of affected sib pairs, the inflation factor simplifies to *γ *= 1 + *F*_*IT *_+ 2*F*_*ST*_. The total numerator can be expressed as the estimated allele frequency difference between cases and controls

$$T=1/(2n_{1)}\sum {\;}_{i:y_{i}=1}X_{i.}-1/\left(2n_{0}\right)\sum {\;}_{i:y_{i}=0}X_{i.}.$$

Under the null hypothesis of no association, its variance can be derived by dividing the sum of alleles within cases into two parts: one for affected sib pairs and one for independent cases, leading to

$$Var_{\gamma }(T)=p(1-p)(\left(m\gamma +n_{1}-m\right)/(2n_{1}^{2})+1/\left(2n_{0}\right)).$$

The inflation *γ *for the allelic *χ*^2^-test *Var*_*γ*=1_(*T*) is defined as *λ *= *Var*_*γ*_*T*/*Var*_*γ*=1_*T*.

### Strategies to determine the correlations *F*_*ST *_and *F*_*IT*_

To estimate *Var*(*T)*, different strategies for determining *F*_*ST *_and *F*_*IT *_were investigated. In strategy I ("no linkage") *F*_*ST *_is directly calculated under the assumptions of no linkage and *F*_*IT *_= 0. Here *F*_*ST *_corresponds to the prior kinship coefficient of a sib pair. *F*_*ST *_= 0.25, since 2*F*_*ST *_is the probability that two alleles from the same parent of a sib pair are IBD. In the two other strategies *F*_*ST *_is estimated to account for regions of linkage where the true *F*_*ST *_is larger than 0.25.

In strategy II ("ANOVA") *F*_*ST *_and *F*_*IT *_are estimated by analysis of variance based on the marker data of the affected sibships at the candidate locus [7]. This strategy has no further assumptions and is based on a partitioning of the total sum of squares into three sums of squares: within individuals, within sibships, and between sibships. Each of them describes the additional variance compared to the lower level in the given order. Because *F*_*ST *_and *F*_*IT *_can be expressed as ratios of variance components, estimates for *F*_*ST *_and *F*_*IT *_can be derived as functions of the sums of squares.

Strategy III ("MULTI") uses a multipoint *F*_*ST *_estimate assuming *F*_*IT *_= 0, requiring genotype information at adjacent markers, e.g., for cases previously analyzed for linkage with these markers. *F*_*ST *_can be directly estimated from the estimated mean number *Y *of alleles IBD within the affected sib pairs. The expectation of *Y *can be expressed as *E(Y) = 2N·2F*_*ST*_, where $2N=\sum {\;}_{k=1}^{K}m_{k}(m_{k}-1)$ is the total number of allelic pairs considered and *2F*_*ST*_, is the probability that such an allele pair is IBD. The estimated number Y of alleles IBD has to be calculated from individual IBD estimates. If there are only affected sib pairs in the data (*N *= *K*), *Y *can be derived from the nonparametric linkage-score (NPL- or Z-score), which is then equivalent to the classical mean test statistic $Z=\left(Y-K\right)/\sqrt{K/2}$. Here the same IBD measure is used as in linkage analysis.

To evaluate the strategies we implemented the test statistic in the computer program R. For strategy I *F*_*ST *_= 0.25, for strategy II *F*_*ST *_was estimated in the ANOVA framework implemented in R, and for strategy III we calculated NPL-scores with Merlin.

### Application to data from a candidate gene study for rheumatoid arthritis

The proposed methods were applied to case-control data from 20 candidate genes for rheumatoid arthritis previously analyzed by Plenge et al. [6]. The 839 cases were from the North American Rheumatoid Arthritis Consortium (NARAC) and include 717 cases from affected sibships and 122 unrelated cases. The 855 unrelated controls were selected from healthy individuals who were enrolled in the New York Cancer Project (NYCP). Because we have to include additional data for strategy III, we only investigated the introduced test statistics based on strategy I, II, and the traditional allelic *χ*^2^-test based on allele frequencies ignoring familial correlations. We compared our results to Plenge et al. [6] who analyzed the same sample with only a few additional individuals.

### Application to the simulated data

Additionally, the SNP genome data from Replicate 1 of the simulated Genetic Analysis Workshop 15 data were analyzed knowing the solutions. The data contain 1500 families of two parents and an affected sib pair and 2000 controls. We calculated our test statistics based on strategies I-III for all 9187 SNPs of the genome scan comparing 3000 cases to 2000 controls. Subsequently, in order to remove true associations, we excluded SNPs in a region around ±3 cM of simulated disease loci to analyze data simulated under the null hypothesis of no association but allowing for linkage. For the remaining SNPs we verified the type I error rate of the test statistics. We also analyzed chromosome 6 containing the major disease locus to concentrate the analysis on a region of known linkage.

## Results

### Results for the candidate gene study for rheumatoid arthritis

Table 1 contains the candidate genes that show a significant association based on the traditional allelic *χ*^2^-test. It shows whether these associations remain significant after accounting for the IBD sharing of the cases. In the ANOVA model *F*_*ST *_is slightly underestimated, being below 0.25. Thus in this example the *p*-values for the "no linkage"-strategy are slightly more conservative than for ANOVA. The variance inflation *λ *of the allelic *χ*^2^-test is estimated around 1.20–1.25. The exact value depends on the strategy of estimating *F*_*ST *_and the number of missing values. By using a significance level of 0.05, all test statistics remain significant with the correct variance estimate. If a Bonferroni corrected significance level of 0.0025 is used, *PTPN22*, *CTLA*, and *SUMO4/rs237025 *(unexpected direction) are significant for the two-sided allelic *χ*^2^-test. For the test statistics accounting for familial correlations, only *PTPN22 *clearly remains significant, *CTLA *is no longer significant and the *p*-value for *SUMO4 *is very close to the significance level.

**Table 1:** Results for selected candidate genes

			GAW data set				
				
	Allele frequency		F_ST_		*p*-value		
						
Gene/marker	Case	Control	ANOVA	ANOVA	no linkage	*χ*^2^-test	*p*-value^a^
*PTPN22/r*s2476601	0.17	0.08	0.25	<0.0001	<0.0001	<0.0001	<0.0001
*CTLA4/*CT60	0.39	0.45	0.23	0.0051	0.0054	0.0019	0.0010
*HAVCR1/*5509_5511delCAA	0.20	0.23	0.22	0.0223	0.0241	0.0117	0.99
*SUM04/r*s237025	0.51	0.46	0.25	0.0022	0.0026	0.0008	0.99
*SUM04/r*s577001	0.39	0.35	0.23	0.0280	0.0323	0.0166	-

^a^One-sided *p*-values for the allelic *χ*^2 ^test given by Plenge et al. If the association was not concordant to previous studies, these *p*-values were set to 0.99 [6].

### Results for the simulated data

**Figure 1:**
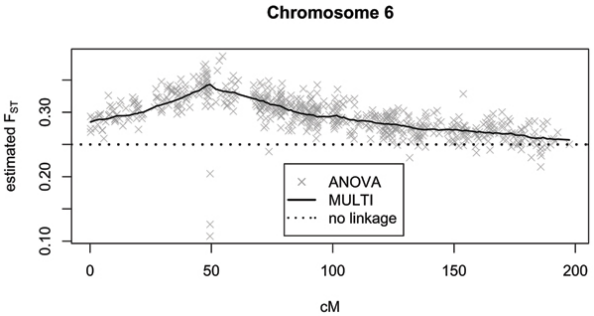
Estimated *F*_*ST *_values for chromosome 6 in the simulated data.

**Figure 2:**
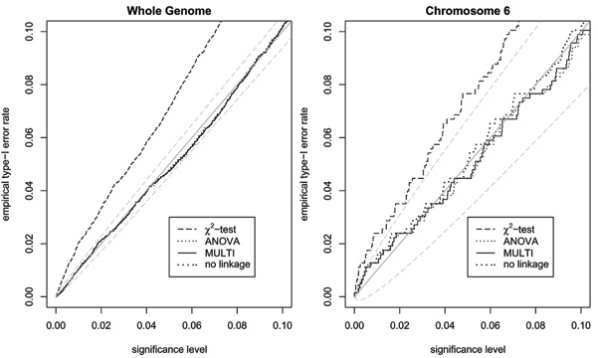
**Observed type I error rates in the simulated data excluding regions of true associations (expected values and 95% confidence bounds in gray).**

Figure 1 shows the estimated *F*_*ST *_values for chromosome 6. As expected, the multipoint *F*_*ST *_estimation is more stable than the single-point ANOVA method. However, even with the single-point method, the *F*_*ST *_estimate is in most cases larger than 0.25, thus accounting for linkage correctly. For the simulated data an *F*_*ST *_value of 0.25 leads to an inflation factor of 1.2, whereas an *F*_*ST *_= 0.3 corresponds to *λ *= 1.24. Because of this small difference between the inflation factors, the method to determine *F*_*ST *_is expected to have only a minor impact on the test statistic. After excluding regions of true associations, 9055 SNPs remained, including 627 out of 674 SNPs on chromosome 6. Figure 2 shows the observed type I error rate for the different test statistics. The results for the entire genome indicate that the allelic *χ*^2^-test is far too liberal. In contrast, the observed type I error rates for the test statistics accounting for familial correlations are all very close to each other within the expected range for all significance levels up to 0.1. The separate analysis of chromosome 6 confirms that even in a region of known linkage there is only a minor difference between the three strategies, with the "no-linkage" being the most liberal.

## Conclusion

If related cases are included in a case-control study, the allelic *χ*^2^-test can lead to an increased rate of false positives, as indicated by the simulations and the real data analysis. All strategies to correct the variance perform quite well and lead to an almost correct type I error rate on the entire genome. In the presence of linkage, test statistics based on estimating the correlations from data are somewhat superior, but a single-point strategy based on the candidate gene data seems sufficient. Moreover, our conclusions are consistent with the simulation results of Bourgain [5], who observed only a minor difference in power between the association test of Slager and Schaid [4] based on IBD estimates and the test of Bourgain et al. [2] based on prior kinship coefficients.

## Competing interests

The author(s) declare that they have no competing interests.
